# Epigenome-wide association study identifies neonatal DNA methylation associated with two-year attention problems in children born very preterm

**DOI:** 10.1038/s41398-024-02841-y

**Published:** 2024-02-28

**Authors:** Marie Camerota, Barry M. Lester, Francisco Xavier Castellanos, Brian S. Carter, Jennifer Check, Jennifer Helderman, Julie A. Hofheimer, Elisabeth C. McGowan, Charles R. Neal, Steven L. Pastyrnak, Lynne M. Smith, Thomas Michael O’Shea, Carmen J. Marsit, Todd M. Everson

**Affiliations:** 1 Department of Psychiatry and Human Behavior, Alpert Medical School of Brown University, Providence, RI USA; 2 Brown Center for the Study of Children at Risk, Alpert Medical School of Brown University and Women and Infants Hospital, Providence, RI USA; 3 Department of Pediatrics, Alpert Medical School of Brown University and Women and Infants Hospital, Providence, RI USA; 4 Department of Child and Adolescent Psychiatry, NYU Grossman School of Medicine, New York, NY USA; 5 Nathan Kline Institute for Psychiatric Research, Orangeburg, NY USA; 6 Department of Pediatrics-Neonatology, Children’s Mercy Hospital, Kansas City, MO USA; 7 Department of Pediatrics, Wake Forest School of Medicine, Winston-Salem, NC USA; 8 Department of Pediatrics, University of North Carolina at Chapel Hill School of Medicine, Chapel Hill, NC USA; 9 Department of Pediatrics, University of Hawaii John A. Burns School of Medicine, Honolulu, HI USA; 10 Department of Pediatrics, Spectrum Health-Helen DeVos Hospital, Grand Rapids, MI USA; 11 Department of Pediatrics, Harbor-UCLA Medical Center, Torrance, CA USA; 12 Gangarosa Department of Environmental Health, Rollins School of Public Health, Emory University, Atlanta, GA USA

**Keywords:** Human behaviour, ADHD

## Abstract

Prior research has identified epigenetic predictors of attention problems in school-aged children but has not yet investigated these in young children, or children at elevated risk of attention problems due to preterm birth. The current study evaluated epigenome-wide associations between neonatal DNA methylation and attention problems at age 2 years in children born very preterm. Participants included 441 children from the Neonatal Neurobehavior and Outcomes in Very Preterm Infants (NOVI) Study, a multi-site study of infants born < 30 weeks gestational age. DNA methylation was measured from buccal swabs collected at NICU discharge using the Illumina MethylationEPIC Bead Array. Attention problems were assessed at 2 years of adjusted age using the attention problems subscale of the Child Behavior Checklist (CBCL). After adjustment for multiple testing, DNA methylation at 33 CpG sites was associated with child attention problems. Differentially methylated CpG sites were located in genes previously linked to physical and mental health, including several genes associated with ADHD in prior epigenome-wide and genome-wide association studies. Several CpG sites were located in genes previously linked to exposure to prenatal risk factors in the NOVI sample. Neonatal epigenetics measured at NICU discharge could be useful in identifying preterm children at risk for long-term attention problems and related psychiatric disorders, who could benefit from early prevention and intervention efforts.

Attention-Deficit/Hyperactivity Disorder (ADHD) is one of the most prevalent mental health disorders in young children [1] and is associated with functional impairment in academic, social, and family settings [2] as well as sizeable social and economic costs [3, 4]. Children born preterm are at higher risk, experiencing rates of ADHD that are 2 to 4 times higher than the general population, with the risk increasing with each decreasing week of gestation at birth [5–7]. Despite this, little is known about the antecedents of attention problems, a predominant characteristic of ADHD, in children born very preterm.

Prior research has described a complex etiology underlying the development of attention problems, with both genetic and environmental factors thought to jointly contribute to risk [8]. More recently, epigenetics has been identified as an important biological domain that could predict risk for attention problems, serving as either a predictive biomarker or a causally implicated biological mechanism [9]. Specifically, the epigenetic mechanism of DNA methylation holds promise as a predictor of attention problems because the methylome is influenced by both genetic and environmental factors, including some of the environmental factors (e.g., smoking, alcohol, adversity, lead) that are implicated in the development of ADHD.

Early studies investigating DNA methylation and ADHD consisted of candidate gene studies that primarily targeted genes involved in the dopaminergic network (e.g., *DRD4*) [10–12]. In recent years epigenome-wide association studies (EWAS) have reported DNA methylation at other genetic loci associated with increased risk for attention problems in children [13–19]. Methylation of the *VIPR2* gene—a gene that codes a receptor for a small neuropeptide with neurotransmitter and neuroendocrine functions—was shown to differentiate between ADHD cases and controls in boys age 7–12 [16], in a sample of twin pairs discordant for ADHD [17], and in the most recent case-control EWAS of approximately 600 children age 7–12 [15]. In prospective, longitudinal studies, DNA methylation at birth has been shown to be associated with later ADHD symptom severity, in genes such as *ZNF544*, *ST3GAL3*, *ERC2,* and *CREB5* [13, 14]. Genetic variation within some of these genes has been implicated in ADHD in prior genome-wide (as opposed to epigenome-wide) association studies [20, 21]. Interestingly, studies with repeated measures of epigenetic data have failed to find concurrent associations between DNAm and ADHD symptoms measured in childhood [13, 14], suggesting DNAm in the neonatal period may be a particularly important predictor of later outcome.

While these prior studies underscore the potential utility of epigenetic studies for understanding the etiology of ADHD, they have not specifically investigated epigenetic precursors to attention problems in children born preterm. Additionally, many prior studies investigated ADHD as a dichotomy (i.e., cases versus controls) rather than measuring symptoms continuously, although the latter approach is gaining popularity [14] perhaps due to its consistency with recent framing of ADHD as a dimensional trait [22, 23]. Finally, prior studies have tended to assess symptoms of ADHD in school-age children, rather than in toddlerhood or early childhood, despite evidence that early attention problems quantified using validated assessments are associated with subsequent attention deficits at school age [24]. The current study aims to address these gaps by conducting an EWAS to examine epigenetic predictors of attention problems at age 2 years in a multi-site study of children born < 30 weeks gestational age (GA).

## Methods

### Participants

Participants were drawn from the Neonatal Neurobehavior and Outcomes in Very Preterm Infants (NOVI) Study, a multi-site study of infants born < 30 weeks GA. Participants were recruited from nine university-affiliated NICUs across six research sites from April 2014 to June 2016. Inclusion criteria were: (1) birth < 30 weeks GA, (2) parental ability to speak English or Spanish, (3) residence within 3 h of the NICU and follow-up clinic. Exclusion criteria included major congenital anomalies, maternal age < 18 years, cognitive impairment, and death. Parents of eligible infants were approached when infants were 31–32 weeks GA or when survival to discharge was deemed likely by the attending neonatologist. Researchers at each site obtained informed consent in line with each institution’s review board. This study followed the Strengthening the Reporting of Observational Studies in Epidemiology (STROBE) guidelines.

Children were included in this analysis if they were enrolled in NOVI, had a neonatal buccal swab collected at NICU discharge, and had attention problems assessed at 24-month follow-up. The majority of infants enrolled in NOVI (651 of 704; 92%) had parental consent for buccal swab collection. Demographic information was collected at enrollment via maternal interview, and information about neonatal health was obtained via standardized medical record abstraction using Vermont-Oxford Network criteria [25].

### Measures

#### Neonatal DNA methylation

Genomic DNA was extracted from buccal swab samples, collected near term-equivalent age, using the Isohelix Buccal Swab system (Boca Scientific), quantified using the Quibit Fluorometer (Thermo Fisher, Waltham, MA, USA) and aliquoted into a standardized concentration for subsequent analyses. DNA samples were plated randomly across 96-well plates and provided to the Emory University Integrated Genomics Core for bisulfite modification using the EZ DNA Methylation Kit (Zymo Research, Irvine, CA), and subsequent assessment of genome-wide DNAm using the Illumina MethylationEPIC Beadarray (Illumina, San Diego, CA) following standardized methods based on the manufacturer’s protocol.

Pre-processing of data followed a previously described workflow [26]. Array data weunderwent Noob normalization [27, 28]. Samples with poor detection p-values or sex-mismatch were excluded. We excluded probes with median detection *p*-values < 0.05, those on the X or Y chromosome, those with single nucleotide polymorphisms (SNP) within the binding region, and those that could cross-hybridize to other regions of the genome [29]. Array data were standardized across Type-I and Type-II probe designs with beta-mixture quantile normalization [30, 31].

We next took steps to decrease multiple testing burden and increase our power to detect meaningful associations. First, we implemented the CoMeBack pipeline [32] to identify co-methylated regions (CMRs) which are clusters of highly-correlated, proximal CpG sites. Principal components analysis is performed for each CMR and the first principal component is assigned to each cluster as a summary of DNAm levels at that CMR. The CoMeBack pipeline identified 73,746 CMRs representing the DNAm of 206,195 CpG sites; 500,128 CpG sites were not included in CMRs and were retained as individual CpG sites. Next, we excluded CpGs or CMRs with low variability (SD < 0.02); sites with low variability are more prone to measurement error and are less likely to result in reproducible findings [33]. To further decrease the likelihood of spurious or non-reproducible findings, we examined each CpG and CMR for outliers and recoded values that fell 3 interquartile ranges (IQR) below the 25^th^ percentile or 3 IQR above the 75^th^ percentile to missing.

After exclusions and data reduction, 452,453 loci (60,917 CMRs and 391,536 CpGs) were available from 542 samples for this study (83% of 651 with buccal swab consent; 77% of entire NOVI cohort). For simplicity in the results, we refer to each loci as a CpG but note where significant results were located in a CMR. These data are accessible through NCBI Gene Expression Omnibus (GEO) via accession series GSE128821.

#### Child Behavior Checklist 1 ½ - 5 years (CBCL)

The CBCL is a parent-report measure of child behavior problems. Caregivers rate the extent to which 99 specific child behaviors apply to their child on a scale of 0 (“Not True”), 1 (“Somewhat or Sometimes True”), or 2 (“Very True or Often True”). Individual items are summed into 7 symptom subscales which can be converted to norm-referenced T-scores (range = 50 to 100). Attention problem T-scores were the primary outcome in this analysis (M = 56.2; SD = 7.43, range = 50 to 80).

#### Covariates

As DNAm levels differ by cell type, estimating cell-type composition of mixed cell samples (e.g., buccal tissue) is important for addressing confounding. We estimated the proportion of epithelial, fibroblast, and immune cells in our buccal tissue using previously developed reference methylomes [34]. As reported in our prior work [35, 36], the majority of our samples were comprised primarily of epithelial cells, with a smaller proportion of immune cells. Given the strong inverse association between epithelial and immune cell proportions in our data, we adjusted all analyses for epithelial cell proportion to address cellular heterogeneity. We also accounted for potential batch effects by adjusting for sample plate.

Besides these technical covariates, we additionally adjusted all EWAS models for study site, infant GA at birth, infant GA at buccal swab (i.e., time between conception and biosample collection), infant sex, and neonatal medical morbidities. In sensitivity analyses, we additionally adjusted for genetic confounding by re-running all models controlling for first-degree relative (e.g., parent, sibling) history of ADHD, as reported on maternal interviews. We also examined maternal prenatal smoking, maternal low socioeconomic status (i.e., Hollingshead level 5), and child birthweight as additional confounders in sensitivity analyses.

#### Statistical analysis

Epigenome-wide analyses were conducted to examine the association of DNAm at each of 452,453 CpG sites and attention problem T-scores. We used generalized estimating equation (GEE) models with robust standard errors to regress CBCL attention problem T-scores (dependent variable) on DNAm at each CpG site, accounting for nesting of children within families and covariates (study site, infant GA at birth, infant GA at buccal swab, infant sex, neonatal medical morbidities, cell type composition [proportion of epithelial cells], and sample plate). P-values were adjusted for multiple testing using the Benjamini-Hochberg false discovery rate (FDR) [37]. CpG sites associated with attention problems within a 5% FDR cutoff were considered significant. For ease of interpretation, we rescaled DNAm at each CpG site by dividing the raw data by the CpG-specific interquartile range (IQR) so that beta coefficients derived from the GEE models can be interpreted as the expected change in attention problem T-scores associated with a change in DNAm from the 25^th^ to the 75^th^ percentile of observed data.

Buccal swabs are a peripheral tissue, whereas the primary mechanistic effects of DNAm on attention problems are likely to be neural. To understand whether the sites we identify in peripheral buccal tissue could be representative of processes occurring in the central nervous system, we investigated whether the methylation levels at our identified CpGs were correlated between brain and buccal samples. For all CpGs significantly associated with attention problems in our EWAS, we estimated the correlation between DNAm of that CpG in brain and buccal tissue using an existing database [38]. To better understand the biological processes underlying the associations between DNAm and attention problems, we additionally conducted gene enrichment analyses using the *gometh* function in the *MissMethyl* package [39] and tested for pathway-based gene set overrepresentation (KEGG and gene ontology [GO] terms). Pathways that were enriched within a 5% FDR were deemed significant. Statistical code for all analyses are available upon request from the first author.

We also examined whether any of the CpGs identified in our analysis annotated to genes that have previously been linked to phenotypic characteristics in genome-wide association studies (GWAS) using the NHGRI-EBI GWAS catalog [40]. Similarly, we examined overlap with published studies in the MRC-IEU EWAS catalog [41]. Finally, we examined whether any of the CpGs or genes identified in the current analysis have been identified in prior EWAS of attention problems in children [13–19].

## Results

### Descriptive statistics

**Fig. 1:**
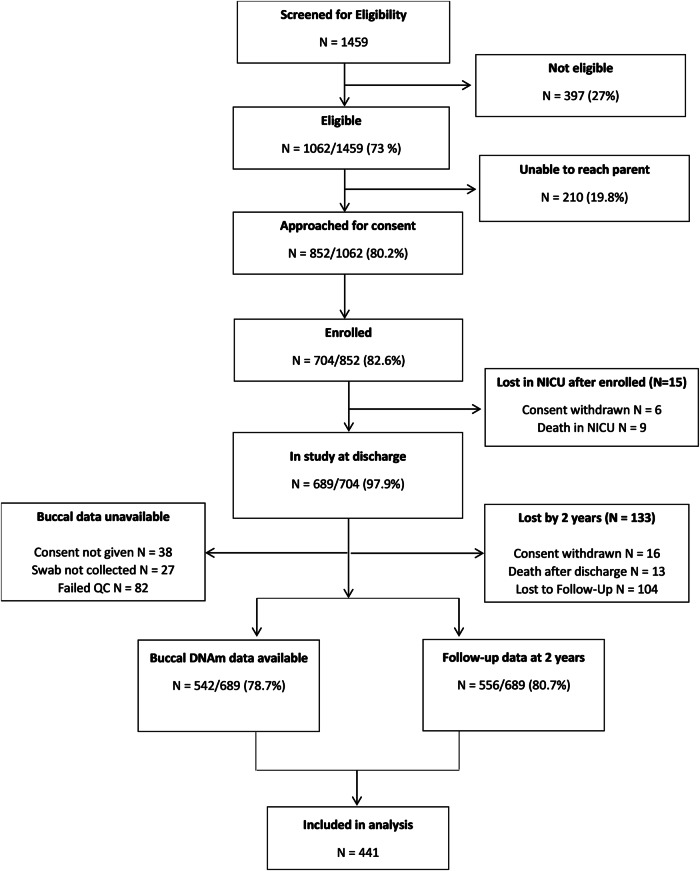
Study flowchart showing participant inclusion and exclusion.

Of the 704 infants enrolled in NOVI, 441 had both buccal swab and CBCL data and were included in these analyses (Fig. 1). The majority of the sample (79%) consisted of singleton births (350 children) with a smaller number of twins (80 children), triplets (3 children), and quadruplets (8 children). Characteristics of the full sample, as well as those included versus excluded in this analysis, are shown in Table 1. Included infants were more likely to be White (48% vs. 32%, *p* < 0.001) and less likely to be multiracial (18% vs. 29%, *p* < 0.001). There were no other maternal or neonatal characteristics that differed between included and excluded participants.

**Table 1:** Demographic and medical characteristics of the sample.

Maternal characteristics	Full Sample (*N* = 617)	Included (*N* = 393)	Excluded (*N* = 224)	
	M (SD) or % (*n*)	M (SD) or % (*n*)	M (SD) or % (*n*)	*p*-value
Minority race or ethnicity	57% (347/606)	52% (205/391)	66% (142/215)	0.002
American Indian/Alaska Native race	0.16% (1/617)	0.25% (1/393)	0% (0/224)	1.00
Asian race	3.7% (23/617)	3.8% (15/393)	3.6% (8/224)	1.00
Native Hawaiian/Other Pacific Islander race	1.3% (8/617)	1% (4/393)	1.8% (4/224)	0.66
Black or African American race	20% (126/617)	21% (81/393)	20% (45/224)	0.96
White race	42% (261/617)	48% (189/393)	32% (72/224)	<0.001
More than one race	22% (136/617)	18% (70/393)	29% (66/224)	<0.001
Unknown/Not reported race	10% (62/617)	8.4% (33/393)	13% (29/224)	0.10
Hispanic/Latino ethnicity	23% (142/617)	21% (83/393)	26% (59/224)	0.17
Ethnicity Unknown	1.3% (8/617)	0% (0/393)	3.6% (8/224)	0.001
Low SES: Hollingshead level 5	9.6% (58/605)	9% (35/391)	11% (23/214)	0.57
Maternal education: < HS/GED	13% (78/604)	15% (57/390)	9.8% (21/214)	0.12
No partner	25% (152/605)	26% (103/391)	23% (49/214)	0.40
**Neonatal characteristics**	**Full Sample (*N*** = **704)**	**Included (*N*** = **441)**	**Excluded (*N*** = **263)**	
Multiple gestations	26% (184/697)	28% (123/440)	24% (61/257)	0.26
Vaginal delivery	29% (201/696)	29% (128/440)	29% (73/256)	0.94
Severe retinopathy of prematurity (ROP)	5.9% (41/697)	5.9% (26/440)	5.8% (15/257)	1.00
Necrotizing enterocolitis/sepsis	18% (128/697)	20% (88/440)	16% (40/257)	0.18
Bronchopulmonary dysplasia (BPD)	51% (357/697)	52% (228/440)	50% (129/257)	0.74
Serious brain injury	13% (92/694)	12% (54/439)	15% (38/255)	0.39
Sex = Male	55% (390/704)	54% (238/441)	58% (152/263)	0.36
GA at birth (weeks)	27.01 (1.91)	27.01 (1.93)	27 (1.89)	0.98
Head circumference (cm)	24.46 (2.43)	24.46 (2.46)	24.48 (2.38)	0.91
GA at NICU discharge (weeks)	40.53 (5.43)	40.35 (5.26)	40.85 (5.72)	0.26
Length of NICU stay (days)	94.16 (44.1)	92.8 (42.9)	96.49 (46.2)	0.30
Birth weight (g)	948.3 (281)	948.9 (285)	947.2 (273)	0.94
Weight at discharge (g)	3014 (905)	2990 (856)	3055 (984)	0.38

*Note*. *GA*, gestational age; *HS*, high school; *GED*, General Equivalency Diploma; *SES*, socioeconomic status. Minority race or ethnicity was defined as any non-White race (e.g., Black, Asian) or ethnicity (e.g., Hispanic and/or Latino/a). Serious brain injury included parenchymal echodensity, periventricular leukomalacia, or ventricular dilation diagnosed via cranial ultrasound.

### EWAS findings

**Fig. 2:**
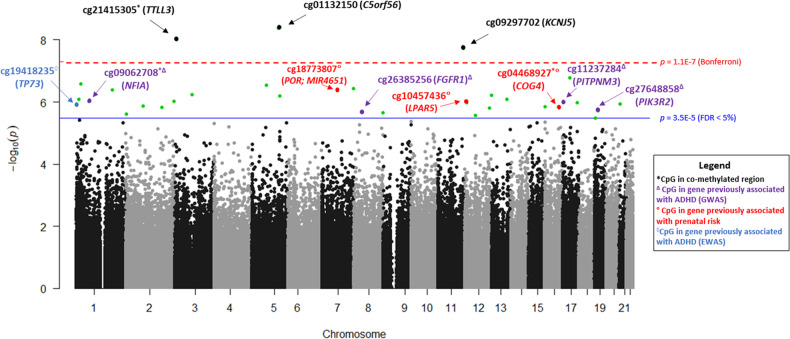
Manhattan plot of epigenetic loci associated with 2 year attention problems. Significant associations (FDR < 5%) are shown above the blue solid line (*p* < 3.5E-5). Bonferroni-significant CpG sites are shown above the red dashed line (*p* < 1.1E-7) and annotated in black. Highlighted in blue (with ^◊^) is one significant CpG site (FDR < 5%) located in a gene whose methylation has previously been shown to be associated with ADHD in a prior EWAS (*TP73*). Highlighted in purple (with ^**Δ**^) are 4 significant CpG sites (FDR < 5%) located in genes that have been shown to be associated with ADHD in prior GWAS studies (*FGFR1, NFIA, PITPNIM3, PIK3R2*). Three CpG sites, highlighted in red (with °), are located in genes we previously found to be associated with prenatal risk in this sample (*POR; MIR4651; COG4; LPAR5*). *Denotes CpG located in co-methylated region (CMR).

DNA methylation at 33 CpG sites was associated with child attention problems (Table 2; Fig. 2). Of these, there were 6 positive associations (i.e., higher DNAm associated with more attention problems) and 27 negative associations (i.e., lower DNAm associated with more attention problems). Of the 33 significant results, 5 were located in CMRs (Table 2). Overall, the associations were small in magnitude: going from the 25^th^ to 75^th^ percentile of DNAm was associated with a 1.3 to 3.2 point change in attention problem T-scores.

**Table 2:** Epigenome-wide association study results for statistically significant CpG sites (FDR < 5%).

CpG / CMR	Location	Gene annotation	Coefficient	Std Error	*p* value (raw)	*p* value (FDR)	Brain-buccal correlation
cg06913365	chr1: 13825255	LRRC38 (Body)	−2.66	0.54	7.99E-07	0.024	0.09
cg08220278	chr1: 180137345	QSOX1 (Body)	−1.55	0.31	3.98E-07	0.020	0.19
cg22727761	chr1: 24286270	PNRC2 (TSS200)	−2.12	0.41	2.61E-07	0.020	0.22
cg19418235^+^	chr1: 3614558	TP73 (TSS200; Body)	−1.57	0.32	1.21E-06	0.025	0.23
cg09062708	chr1: 61649907-61649973	NFIA (Body)	−1.50	0.31	9.33E-07	0.024	−0.02
cg03355952	chr2: 179316072	PRKRA (TSS1500; TSS200); DFNB59 (TSS200); MIR548N (Body)	−1.81	0.38	1.51E-06	0.026	0.15
cg09560533^+^	chr2: 241776193		1.59	0.32	9.31E-07	0.024	−0.25
cg02843332	chr2: 3283237	TSSC1 (Body)	1.43	0.30	2.44E-06	0.036	0.16
cg08976687	chr2: 85515537	TCF7L1 (Body)	−1.68	0.35	1.34E-06	0.025	0.02
cg01807408	chr3: 87137933		2.18	0.44	5.69E-07	0.024	−0.32
cg21415305	chr3: 9851855-9851862	TTLL3 (TSS200)	−1.81	0.31	9.66E-09	0.002	0.39
cg01132150	chr5: 131782489	C5orf56 (Body)	−1.98	0.34	4.26E-09	0.002	−0.15
cg01277890	chr5: 138731822	LOC389333 (TSS1500)	−2.29	0.46	6.24E-07	0.024	0.40
cg25109393	chr5: 73936428-73936437	ENC1 (1stExon; 5’UTR)	−2.08	0.40	2.91E-07	0.020	0.45*
cg05182265	chr7: 156933206	UBE3C (Body)	−2.97	0.59	3.72E-07	0.020	0.86**
cg18773807	chr7: 75543705	POR (TSS1500); MIR4651 (TSS1500)	−2.42	0.48	3.86E-07	0.020	0.13
cg10020385	chr8: 145159706	MAF1 (1stExon; 5’UTR); SHARPIN (TSS1500)	−3.20	0.68	2.20E-06	0.033	0.70**
cg26385256^+^	chr8: 38326334	FGFR1 (5’UTR; 1stExon)	−1.89	0.40	2.12E-06	0.033	0.10
cg09297702	chr11: 128784689	KCNJ5 (Body)	−1.83	0.32	1.85E-08	0.003	0.11
cg13717333^+^	chr12: 122459966	BCL7A (1stExon; 5’UTR)	−2.10	0.44	1.57E-06	0.026	0.23
cg26076948^+^	chr12: 132892417	GALNT9 (Body)	1.70	0.34	6.15E-07	0.024	−0.23
cg02134355	chr12: 53675972	ESPL1 (Body)	1.62	0.35	2.65E-06	0.037	0.11
cg10457436	chr12: 6745871	LPAR5 (TSS1500)	−1.74	0.36	9.54E-07	0.024	0.16
cg11932091	chr12: 8717391-8717487		−2.40	0.49	1.07E-06	0.024	0.15
cg12228863	chr13: 95069222		2.44	0.50	8.30E-07	0.024	0.04
cg04999580	chr16: 3551846	CLUAP1 (Body)	−2.31	0.48	1.39E-06	0.025	0.24
cg04468927	chr16: 70514664-70514920	COG4 (3’UTR)	−1.59	0.33	1.41E-06	0.025	0.05
cg20139664	chr17: 36628961	ARHGAP23 (Body)	−1.73	0.33	1.66E-07	0.019	0.07
cg11237284	chr17: 6458238	PITPNM3 (Body)	−1.67	0.34	9.88E-07	0.024	0.14
cg22514284	chr17: 72754345	SLC9A3R1 (Body)	−1.44	0.29	1.05E-06	0.024	0.03
cg27648858	chr19: 18266834	PIK3R2 (Body)	−1.46	0.31	1.91E-06	0.031	−0.29
cg14798653	chr19: 6475497	DENND1C (Body)	−1.33	0.28	3.24E-06	0.044	−0.14
cg05076365	chr21: 17011727		−1.76	0.36	1.14E-06	0.025	−0.06

*Note*. The coefficient represents the expected increase or decrease in CBCL T-scores associated with an increase of DNAm from the 25^th^ to 75^th^ percentile. CpGs listed with a range of genomic position are located in co-methylated regions (CMRs). *p < 0.05, **p < 0.01. ^+^Denotes CpGs that were no longer significantly associated with CBCL T-scores after adjustment for familial confounding.

There were significant, positive brain-buccal correlations for 3 of the 33 identified CpG sites (cg25109393, cg05182265, cg10020385). These correlations were moderate to large in magnitude (*r* = 0.45 to 0.86, all *p* < 0.05). After FDR correction, we failed to identify any significantly enriched pathways using either the KEGG or GO methods.

There were several relevant phenotypes and traits associated with the genes annotated to the significant CpG sites from our EWAS (Table 3). Four CpGs (cg26385256, cg09062708, cg27648858, cg11237284) were located in genes that have been found to be associated with ADHD in prior GWAS (*FGFR1, NFIA, PITPNIM3, PIK3R2*). Three CpGs (cg18773807, cg04468927, cg10457436) were located in genes we previously found to be associated with cumulative prenatal risk in this sample (*POR; MIR4651; COG4; LPAR5*) [36]. Three of the 33 CpGs met a strict Bonferroni adjustment for multiple testing (cg21415305, cg01132150, cg09297702). These CpGs are annotated to the *TTLL3*, *C5orf56*, and *KCNJ5* genes. A comparison of our findings with the EWAS catalog (Table 3) uncovered that two of our significant CpGs (cg05182265, cg27648858) have previously been associated with maternal prenatal risk factors (i.e., smoking and hypertensive disorders of pregnancy).

**Table 3:** CpGs associated with child attention problems (FDR < 5%) are linked to genes, exposures, and outcomes in the GWAS and EWAS Catalog.

CpG	Location	Gene annotation	GWAS Catalog Hits	GWAS Catalog Entries	EWAS Catalog Hits	EWAS Catalog Entries
cg06913365	chr1: 13825255	LRRC38	6	Blood pressure	1	Incident Type 2 Diabetes
cg08220278	chr1: 180137345	QSOX1	15	Blood pressure, anorexia nervosa	—	
cg22727761	chr1: 24286270	PNRC2	—		1	HIV infection
cg19418235	chr1: 3614558	TP73	11	Leukemia, visceral adipose tissue measurement, waist-hip ratio, lung carcinoma	—	
cg09062708	chr1: 61649907	NFIA	274	Bipolar disorder, risk-taking behavior, educational attainment, brain measurement, mathematical ability, ADHD, substance abuse, antisocial behavior, autism spectrum disorder, anxiety, preterm birth	9	Tissue, GA, Age, Rheumatoid arthritis, Alzheimer’s disease break stage
cg03355952	chr2: 179316072	PRKRA	6	High density lipoprotein cholesterol measurement, atopic eczema, psoriasis	—	
		DFNB59	—			
		MIR548N	—			
cg09560533	chr2: 241776193	—	—		1	Tissue
cg02843332	chr2: 3283237	TSSC1	—		—	
cg08976687	chr2: 85515537	TCF7L1	51	Blood pressure, cervical carcinoma	4	Tissue, age
cg01807408	chr3: 87137933	—			3	Tissue, age, sex
cg21415305	chr3: 9851855	TTLL3	3	Educational attainment	3	Schizophrenia, Progressive supranuclear palsy
cg01132150	chr5: 131782489	C5orf56	—		7	Tissue, GA, Age
cg01277890	chr5: 138731822	LOC389333	—		4	Tissue, age, incident COPD, lung cancer
cg25109393	chr5: 73936428	ENC1	4	Aging, cognition	2	Tissue, HIV infection
cg05182265	chr7: 156933206	UBE3C	35	Type 2 diabetes mellitus, whole brain volume, response to antidepressant, body mass index, systolic blood pressure	6	Tissue, age, GA, maternal smoking in pregnancy
cg18773807	chr7: 75543705	POR	54	Body height, mathematical ability, non-high-density lipoprotein cholesterol measurement	1	Protein levels
		MIR4651	—			
cg10020385	chr8: 145159706	MAF1	2	Mean corpuscular hemoglobin concentration	1	Atopy
		SHARPIN	12	Asthma, hippocampal volume, cortical thickness, Alzheimer’s disease		
cg26385256	chr8: 38326334	FGFR1	66	Body mass index, schizophrenia, cortical thickness, autism spectrum disorder, bipolar disorder, ADHD, response to cognitive behavioral therapy, type 2 diabetes mellitus, birth weight, brain measurement, antisocial behavior, substance abuse	5	Age, pancreatic ductal adenocarcinoma, rheumatoid arthritis
cg09297702	chr11: 128784689	KCNJ5	37	Blood pressure, resting heart rate	—	
cg13717333	chr12: 122459966	BCL7A	37	Body mass index, body weight, educational attainment	2	Age, Tissue
cg26076948	chr12: 132892417	GALNT9	24	Body mass index, antisaccade response, cardiovascular disease, alcohol consumption	2	Age
cg02134355	chr12: 53675972	ESPL1	7	Body mass index	—	
cg10457436	chr12: 6745871	LPAR5	17	High density lipoprotein cholesterol measurement	5	Tissue, age, GA, birthweight
cg11932091	chr12: 8717391	—	—		4	Age, tissue, GA
cg12228863	chr13: 95069222	—	—		—	
cg04999580	chr16: 3551846	CLUAP1	20	Body mass index, educational attainment, type 2 diabetes mellitus, cardiovascular disease	—	
cg04468927	chr16: 70514664	COG4	6	Body mass index, body weight	2	Tissue, GA
cg20139664	chr17: 36628961	ARHGAP23	16	Reaction time, brain measurement	—	
cg11237284	chr17: 6458238	PITPNM3	17	Blood pressure, educational attainment, reaction time, ADHD, substance abuse, antisocial behavior	1	Protein levels
cg22514284	chr17: 72754345	SLC9A3R1	25	White blood cell count	—	
cg27648858	chr19: 18266834	PIK3R2	12	Intelligence, ADHD, autism spectrum disorder	7	Tissue, clear cell renal carcinoma, GA, age, hypertensive disorders of pregnancy
cg14798653	chr19: 6475497	DENND1C	3	Brain measurement	5	Tissue, clear cell carcinoma, age, Alzheimer’s disease braak stage, Braak stage
cg05076365	chr21: 17011727	—	—		1	Incident liver cirrhosis

We examined whether any of the CpGs identified in our study were associated with methylation quantitative trait loci (mQTL) using the GoDMC database [42]. We found that 4 CpGs (cg01807408, cg01132150, cg05182265, cg11932091) have previously been identified as mQTLs.

### Sensitivity Analyses

To address the potential for genetic confounding, we conducted sensitivity analyses that additionally adjusted for first-degree relative (e.g., parent, sibling) history of ADHD. Of the 33 CpGs identified as significant in the main EWAS, 28 remained significant (FDR < 5%) after this additional adjustment. The CpGs no longer significant after this additional adjustment are noted in Table 2 with a symbol (^+^).Overall, additional adjustment for familial confounding did not explain the majority of our significant findings.

We also examined the potential confounding effect of three additional covariates: maternal prenatal smoking, maternal low socioeconomic status (Hollingshead level 5), and child birthweight. Inclusion of these additional covariates did not substantively change the reported results. All 33 CpGs identified as significant in the main EWAS remained significant (FDR < 5%) after additional adjustment. Full results from all sensitivity models are presented as Supplementary Material.

## Discussion

The purpose of this study was to conduct an EWAS to identify neonatal DNAm predictors of attention problems in infants born very preterm. We found 33 CpGs that were significantly associated with age 2 attention problems. Several of these CpGs annotated to genes previously found to be associated with ADHD. This study extends prior research by showing associations between DNAm at NICU discharge and attention problems, measured dimensionally, in toddlerhood, and is also the first EWAS investigating attention problems in children born very preterm.

Prior EWAS investigating attention problems, though not conducted specifically with preterm populations, have similarly found epigenetic signatures at birth associated with later ADHD diagnosis or symptom severity [13–19]. One of the CpGs identified in the current study (cg19418235) is located in the *TP73* gene. Another CpG located in this gene (cg06996273) was identified in a prior study comparing DNAm of twin pairs discordant for ADHD diagnosis [17]. In the prior study, ADHD cases had higher DNAm of this CpG compared to controls, whereas in the current study, we found that lower DNAm of our CpG was associated with more parent-rated attention problems. The different direction of associations between these studies may be due to the different locations of these CpGs: cg19418235 is located 0–200 bases upstream of the transcription start site whereas cg06996273 is located in the gene body. While lower DNAm in the transcription start site is typically associated with increased transcriptional activity, the inverse is often true for gene body methylation, where DNAm is more frequently positively associated with transcription. Thus, the different directions of association between these two studies, at two different CpG, may actually be reflective of similar epigenetic regulation of the *TP73* gene. The *TP73* gene (tumor protein p73) encodes one of a family of transcription factors involved in cellular response to development and stress, including apoptotic signaling in response to DNA damage. Although genetic variation in TP73 has been associated with various types of cancer [43, 44], differential methylation of this gene is not well studied and its potential role during early development is not clear.

There were no other CpGs or genes found in the current analysis that overlapped with previous ADHD or attention EWAS. This may be due to differences in the tissue type used (prior studies have not investigated DNAm from buccal swabs), outcome measures (attention problems measured dimensionally versus ADHD diagnosis or ADHD symptom severity), age at outcome (age 2 versus school-age children), unsystematic differences due to chance findings from limited study sample sizes, and our specific investigation of children born < 30 weeks GA. Our choice of covariates compared to prior studies may also have contributed to differences in our findings. For example, we controlled for GA because it has been shown to be associated with both attention problems and patterns of DNA methylation. By controlling for GA, we avoid confounding by this factor but also limit our ability to identify CpG sites that could explain associations between GA and attention problems. While other studies have included additional covariates such as child age [15] we chose not to control for age as our outcome assessments were conducted within a relatively narrow age window.

Considering overlap with genetic (rather than epigenetic) studies, four of the CpGs we found to be associated with attention problems in our study were located in genes that have been linked to ADHD in prior GWAS (*FGFR1*, *NFIA*, *PIK3R2*, *PITPNM3*) [45, 46]. In our study, increased DNAm at all four CpG sites was associated with lower attention problem scores. Interestingly, the CpG located in *FGFR1* (cg26385256) was no longer significant after controlling for family history of ADHD. Another one of these CpGs (cg11237284) was located proximal (i.e., 500 bases upstream) to the ADHD-associated SNP (rs1105916) in *PITPNM3*.This overlap in findings from the current and prior EWAS and GWAS studies suggests that both genetic and epigenetic processes likely contribute to risk for attention problems, though their relative contributions is not yet known. Our mQTL search showed that four of our identified CpGs may be mQTLs. Thus, the methylation signals we found in some of our CpGs could represent both genetic and environmental influences on ADHD. We use caution in interpreting these mQTL findings given that the mQTL search was conducted using a database developed in a different tissue type (blood) and age range (primarily adults) compared to the current study.

We have previously conducted EWAS in this sample to investigate epigenetic associations with prenatal risk factors [36], neonatal neurobehavior [35, 47], and neonatal medical morbidities [26]. Interestingly, we found overlap in one specific CpG (cg18773807, annotated to *POR* and *MIR4651*) and two additional genes (*COG4; LPAR5*) that we previously found to be associated with cumulative prenatal risk [36]. The direction of associations for these overlapping findings suggest that an increase in prenatal risk is associated with decreased DNAm at NICU discharge, which in turn in associated with higher attention problem T-scores at age 2 years. One additional CpG (cg05182265) has previously been identified as differentially methylated in children exposed to prenatal maternal smoking [48] a putative risk factor for the development of ADHD [8]. These results are intriguing as they suggest that neonatal DNAm may be one mechanism underlying the well-documented links between prenatal environmental conditions and attention problems in children (for a meta-analysis, see Kim [49]). The majority of these overlapping genes (*POR*, *COG4, LPAR5*) have also previously been linked to markers of physical health and cognitive ability [50, 51].

Our findings are consistent with a growing body of literature linking both genetic and epigenetic variability to differences in attention-related phenotypes, whether measured as dimensional traits, disease symptoms, or ADHD diagnosis. It is important to consider the current findings in the context of our study’s limitations. First, although measuring attention problems in toddlerhood could open the door for early detection of children at higher risk for later impairment, we are not yet able to pinpoint children in our sample who will go on to have persistent attention problems or who will go on to receive an ADHD diagnosis. We also used a single caregiver report of attention problems, which may not be as reliable as having multiple informants or objective assessments. However, as our longitudinal study is ongoing, eventually we will have objective assessment data alongside reported ADHD diagnosis. At that point we plan to investigate whether the neonatal DNAm signal persists or whether there are specific CpGs implicated in later, persistent, and/or clinically relevant attention problems. Second, our investigation of a sample of children born < 30 weeks GA is a unique component of this study, as these children are both understudied and at increased risk for attention problems. As such, we cannot say whether the CpGs identified in this study would be expected to be associated with attention problems in other populations of children or are unique to prematurity. The uniqueness of our sample also means we were unable to identify an appropriate replication dataset. Therefore, further study into the epigenetic predictors of attention problems in early childhood, in both low- and high-risk populations, is warranted. A third limitation is that our DNAm data were obtained using buccal swabs, whereas the tissue that is likely to be causally implicated in attention-related phenotypes is located in the brain. We also observed few significant brain-buccal correlations in the identified CpGs from this study, though the database we used to investigate these correlations was based on a small number of highly selected patients (i.e., those undergoing surgery for epilepsy) with a great degree of variability in patient age and brain tissue location [38]. Nonetheless, it is worth noting that the biological pathways leading from differential DNAm of the identified CpGs to attention problems cannot be parsed out in the current study, nor can we infer causality. Importantly, identification of DNAm loci within buccal cells that are linked to attention problems could be more practically useful for future screening or translation efforts since peripheral tissues (unlike brain tissue) are easily accessible. Future studies that take a multi-omics approach (e.g., adding transcriptomics and/or proteomics) might move the field closer to understanding the underlying biological mechanisms, but these methods remain analytically- and resource-intensive in practice. Finally, although we tested the role of family history of ADHD as an additional covariate, our study currently lacks genomic data, a potentially important source of unmeasured confounding that should be further explored.

In summary, we found DNAm at NICU discharge predicted attention problems at age 2 in a large sample of children born very preterm. Further research should be done to investigate whether the same CpGs or genes remain associated with attention problems measured later in development as well as with formal diagnosis of ADHD in this population. Understanding how changes in DNAm predict later attention problems or attention-related trajectories is another critical next step. This information could be useful in identifying preterm children at risk for later ADHD, who could benefit from additional monitoring and/or targeted early intervention.

### Supplementary information

Supplemental Tables and Figures

Supplemental Materials (Full EWAS Results)

## Data Availability

Data are accessible through NCBI Gene Expression Omnibus (GEO) via accession series GSE128821. Statistical code for all analyses are available upon request from the first author.

## References

[1] Polanczyk G, de Lima MS, Horta BL, Biederman J, Rohde LA (2007). The Worldwide Prevalence of ADHD: A Systematic Review and Metaregression Analysis. AJP.

[2] Barkley RA (1997). Behavioral inhibition, sustained attention, and executive functions: Constructing a unifying theory of ADHD. Psychological Bull.

[3] Leibson CL (2001). Use and Costs of Medical Care for Children and Adolescents With and Without Attention-Deficit/Hyperactivity Disorder. JAMA.

[4] Matza LS, Paramore C, Prasad M (2005). A review of the economic burden of ADHD. Cost Eff Resour Alloc.

[5] Anderson PJ, De Luca CR, Hutchinson E, Spencer-Smith MM, Roberts G, Doyle LW (2011). Attention Problems in a Representative Sample of Extremely Preterm/Extremely Low Birth Weight Children. Developmental Neuropsychol.

[6] Franz AP, Bolat GU, Bolat H, Matijasevich A, Santos IS, Silveira RC (2018). Attention-Deficit/Hyperactivity Disorder and Very Preterm/Very Low Birth Weight: A Meta-analysis. Pediatrics.

[7] Sucksdorff M, Lehtonen L, Chudal R, Suominen A, Joelsson P, Gissler M (2015). Preterm Birth and Poor Fetal Growth as Risk Factors of Attention-Deficit/Hyperactivity Disorder. Pediatrics.

[8] Thapar A, Cooper M, Eyre O, Langley K (2013). Practitioner Review: What have we learnt about the causes of ADHD?. J Child Psychol Psychiatry.

[9] Cecil CAM, Nigg JT (2022). Epigenetics and ADHD: Reflections on Current Knowledge, Research Priorities and Translational Potential. Mol Diagn Ther.

[10] Dadds MR, Schollar-Root O, Lenroot R, Moul C, Hawes DJ (2016). Epigenetic regulation of the DRD4 gene and dimensions of attention-deficit/hyperactivity disorder in children. Eur Child Adolesc Psychiatry.

[11] van Mil NH, Steegers-Theunissen RPM, Bouwland-Both MI, Verbiest MMPJ, Rijlaarsdam J, Hofman A (2014). DNA methylation profiles at birth and child ADHD symptoms. J Psychiatr Res.

[12] Xu Y, Chen XT, Luo M, Tang Y, Zhang G, Wu D (2015). Multiple epigenetic factors predict the attention deficit/hyperactivity disorder among the Chinese Han children. J Psychiatr Res.

[13] Walton E, Pingault JB, Cecil CAM, Gaunt TR, Relton CL, Mill J (2017). Epigenetic profiling of ADHD symptoms trajectories: a prospective, methylome-wide study. Mol Psychiatry.

[14] Neumann A, Walton E, Alemany S, Cecil C, González JR, Jima DD (2020). Association between DNA methylation and ADHD symptoms from birth to school age: a prospective meta-analysis. Transl Psychiatry.

[15] Mooney MA, Ryabinin P, Wilmot B, Bhatt P, Mill J, Nigg JT (2020). Large epigenome-wide association study of childhood ADHD identifies peripheral DNA methylation associated with disease and polygenic risk burden. Transl Psychiatry.

[16] Wilmot B, Fry R, Smeester L, Musser ED, Mill J, Nigg JT (2016). Methylomic analysis of salivary DNA in childhood ADHD identifies altered DNA methylation in *VIPR2*. J Child Psychol Psychiatr.

[17] Chen YC, Sudre G, Sharp W, Donovan F, Chandrasekharappa SC, Hansen N (2018). Neuroanatomic, epigenetic and genetic differences in monozygotic twins discordant for attention deficit hyperactivity disorder. Mol Psychiatry.

[18] Wang Y, Qian M, Tang D, Herbstman J, Perera F, Wang S A powerful and flexible weighted distance-based method incorporating interactions between DNA methylation and environmental factors on health outcomes. Hancock J, ed. *Bioinformatics*. 2020;**36**:653-9. 10.1093/bioinformatics/btz630 10.1093/bioinformatics/btz630 PMC7523680 31504174

[19] Goodman SJ, Burton CL, Butcher DT, Siu MT, Lemire M, Chater-Diehl E (2020). Obsessive-compulsive disorder and attention-deficit/hyperactivity disorder: distinct associations with DNA methylation and genetic variation. J Neurodev Disord.

[20] Demontis D, Walters RK, Martin J, Mattheisen M, Als TD, Agerbo E (2019). Discovery of the first genome-wide significant risk loci for attention deficit/hyperactivity disorder. Nat Genet.

[21] Lasky-Su J, Neale BM, Franke B, Anney RJL, Zhou K, Maller JB (2008). Genome-wide association scan of quantitative traits for attention deficit hyperactivity disorder identifies novel associations and confirms candidate gene associations. Am J Med Genet B Neuropsychiatr Genet.

[22] Willcutt EG, Nigg JT, Pennington BF, Solanto MV, Rohde LA, Tannock R (2012). Validity of DSM-IV attention deficit/hyperactivity disorder symptom dimensions and subtypes. J Abnorm Psychol.

[23] Nigg JT, Sibley MH, Thapar A, Karalunas SL (2020). Development of ADHD: Etiology, Heterogeneity, and Early Life Course. Annu Rev Dev Psychol.

[24] Finsaas MC, Bufferd SJ, Dougherty LR, Carlson GA, Klein DN (2018). Preschool psychiatric disorders: homotypic and heterotypic continuity through middle childhood and early adolescence. Psychol Med.

[25] Vermont Oxford Network. *Manual of Operations: Part 2. Data Definitions and Infant Data Forms*. 2018; Vermont Oxford Network.

[26] Everson TM, O’Shea TM, Burt A, Hermetz K, Carter BS, Helderman J (2020). Serious neonatal morbidities are associated with differences in DNA methylation among very preterm infants. Clin Epigenet.

[27] Liu J, Siegmund KD (2016). An evaluation of processing methods for HumanMethylation450 BeadChip data. BMC Genomics.

[28] Aryee MJ, Jaffe AE, Corrada-Bravo H, Ladd-Acosta C, Feinberg AP, Hansen KD (2014). Minfi: a flexible and comprehensive Bioconductor package for the analysis of Infinium DNA methylation microarrays. Bioinformatics.

[29] Pidsley R, Zotenko E, Peters TJ, Lawrence MG, Risbridger GP, Molloy P (2016). Critical evaluation of the Illumina MethylationEPIC BeadChip microarray for whole-genome DNA methylation profiling. Genome Biol.

[30] Pidsley R, Y Wong CC, Volta M, Lunnon K, Mill J, Schalkwyk LC (2013). A data-driven approach to preprocessing Illumina 450K methylation array data. BMC Genomics.

[31] Teschendorff AE, Marabita F, Lechner M, Bartlett T, Tegner J, Gomez-Cabrero D (2013). A beta-mixture quantile normalization method for correcting probe design bias in Illumina Infinium 450 k DNA methylation data. Bioinformatics.

[32] Gatev E, Gladish N, Mostafavi S, Kobor MS (2020). CoMeBack: DNA methylation array data analysis for co-methylated regions. Bioinformatics.

[33] Logue MW, Smith AK, Wolf EJ, Maniates H, Stone A, Schichman SA (2017). The correlation of methylation levels measured using Illumina 450K and EPIC BeadChips in blood samples. Epigenomics.

[34] Zheng SC, Webster AP, Dong D, Feber A, Graham DG, Sullivan R (2018). A novel cell-type deconvolution algorithm reveals substantial contamination by immune cells in saliva, buccal and cervix. Epigenomics.

[35] Everson TM, Marsit CJ, Michael O’Shea T, Burt A, Hermetz K, Carter BS (2019). Epigenome-wide analysis identifies genes and pathways linked to neurobehavioral variation in preterm infants. Sci Rep..

[36] Camerota M, Graw S, Everson TM, McGowan EC, Hofheimer JA, O’Shea TM (2021). Prenatal risk factors and neonatal DNA methylation in very preterm infants. Clin Epigenet.

[37] Benjamini Y, Hochberg Y (1995). Controlling the false discovery rate: a practical and powerful approach to multiple testing. J R Stat Soc: Ser B (Methodol).

[38] Braun PR, Han S, Hing B, Nagahama Y, Gaul LN, Heinzman JT (2019). Genome-wide DNA methylation comparison between live human brain and peripheral tissues within individuals. Transl Psychiatry.

[39] Phipson B, Maksimovic J, Oshlack A (2016). missMethyl: an R package for analyzing data from Illumina’s HumanMethylation450 platform. Bioinformatics.

[40] MacArthur J, Bowler E, Cerezo M, Gil L, Hall P, Hastings E (2017). The new NHGRI-EBI Catalog of published genome-wide association studies (GWAS Catalog). Nucleic acids Res.

[41] Battram T, Yousefi P, Crawford G, Prince C, Sheikhali Babaei M, Sharp G (2022). The EWAS Catalog: a database of epigenome-wide association studies. Wellcome Open Res.

[42] Min JL, Hemani G, Hannon E, Dekkers KF, Castillo-Fernandez J, Luijk R (2021). Genomic and phenotypic insights from an atlas of genetic effects on DNA methylation. Nat Genet.

[43] Lv H, Zhang M, Shang Z, Li J, Zhang S, Lian D (2017). Genome-wide haplotype association study identify the FGFR2 gene as a risk gene for acute myeloid leukemia. Oncotarget.

[44] Brandes N, Linial N, Linial M (2021). Genetic association studies of alterations in protein function expose recessive effects on cancer predisposition. Sci Rep.

[45] Rao S, Baranova A, Yao Y, Wang J, Zhang F (2022). Genetic relationships between attention-deficit/hyperactivity disorder, autism spectrum disorder, and intelligence. Neuropsychobiology.

[46] Karlsson Linnér R, Mallard TT, Barr PB, Sanchez-Roige S, Madole JW, Driver MN (2021). Multivariate analysis of 1.5 million people identifies genetic associations with traits related to self-regulation and addiction. Nat Neurosci.

[47] Aghagoli G, Sheinkopf SJ, Everson TM, Marsit CJ, Lee H, Burt AA (2021). Epigenome-wide analysis identifies genes and pathways linked to acoustic cry variation in preterm infants. Pediatr Res.

[48] Joubert BR, Felix JF, Yousefi P, Bakulski KM, Just AC, Breton C (2016). DNA methylation in newborns and maternal smoking in pregnancy: genome-wide consortium meta-analysis. Am J Hum Genet.

[49] Kim JH, Kim JY, Lee J, Jeong GH, Lee E, Lee S (2020). Environmental risk factors, protective factors, and peripheral biomarkers for ADHD: an umbrella review. Lancet Psychiatry.

[50] Zhu Z, Guo Y, Shi H, Liu CL, Panganiban RA, Chung W (2020). Shared genetic and experimental links between obesity-related traits and asthma subtypes in UK Biobank. J Allergy Clin Immunol.

[51] Lee JJ, Wedow R, Okbay A, Kong E, Maghzian O, Zacher M (2018). Gene discovery and polygenic prediction from a genome-wide association study of educational attainment in 1.1 million individuals. Nat Genet.

