# Supplementary material for: Epigenome-wide association study identifies neonatal DNA methylation associated with two-year attention problems in children born very preterm
Source: Transl Psychiatry. 2024 Feb 28;14:126. doi: 10.1038/s41398-024-02841-y (PMC10902402; doi:10.1038/s41398-024-02841-y)
Supplement: Supplementary file 1 — Supplemental Tables and Figures [file 41398_2024_2841_MOESM1_ESM.docx]

|  | ***Main Model*** | | | | ***Sensitivity Analysis***  ***(Family history ADHD)*** | | | |
| --- | --- | --- | --- | --- | --- | --- | --- | --- |
| **CpG** | **Coefficient** | **Std Error** | ***p* value (raw)** | ***p* value (FDR)** | **Coefficient** | **Std Error** | ***p* value (raw)** | ***p* value (FDR)** |
| cg01132150 | -1.98 | 0.34 | 4.26E-09 | 0.00193 | -1.98 | 0.43 | 4.24E-06 | 0.00007 |
| cg21415305 | -1.81 | 0.31 | 9.66E-09 | 0.00219 | -1.43 | 0.39 | 2.11E-04 | 0.00077 |
| cg09297702 | -1.83 | 0.32 | 1.85E-08 | 0.00280 | -1.80 | 0.45 | 5.73E-05 | 0.00038 |
| cg20139664 | -1.73 | 0.33 | 1.66E-07 | 0.01878 | -1.64 | 0.43 | 1.39E-04 | 0.00065 |
| cg18773807 | -2.42 | 0.48 | 3.86E-07 | 0.02003 | -1.85 | 0.70 | 8.30E-03 | 0.01305 |
| cg22727761 | -2.12 | 0.41 | 2.61E-07 | 0.02003 | -1.88 | 0.56 | 7.25E-04 | 0.00189 |
| cg08220278 | -1.55 | 0.31 | 3.98E-07 | 0.02003 | -1.39 | 0.39 | 4.11E-04 | 0.00136 |
| cg05182265 | -2.97 | 0.59 | 3.72E-07 | 0.02003 | -3.00 | 0.71 | 2.38E-05 | 0.00020 |
| cg25109393 | -2.08 | 0.40 | 2.91E-07 | 0.02003 | -1.66 | 0.56 | 2.77E-03 | 0.00508 |
| *cg26076948* | *1.70* | 0.34 | *6.15E-07* | *0.02353* | *0.77* | 0.44 | *8.02E-02* | *0.08535* |
| cg01807408 | 2.18 | 0.44 | 5.69E-07 | 0.02353 | 2.05 | 0.61 | 7.46E-04 | 0.00189 |
| cg01277890 | -2.29 | 0.46 | 6.24E-07 | 0.02353 | -2.58 | 0.59 | 1.11E-05 | 0.00012 |
| cg10457436 | -1.74 | 0.36 | 9.54E-07 | 0.02424 | -1.03 | 0.45 | 2.19E-02 | 0.03145 |
| cg22514284 | -1.44 | 0.29 | 1.05E-06 | 0.02424 | -1.12 | 0.35 | 1.54E-03 | 0.00298 |
| cg12228863 | 2.44 | 0.50 | 8.30E-07 | 0.02424 | 1.58 | 0.71 | 2.67E-02 | 0.03584 |
| cg11237284 | -1.67 | 0.34 | 9.88E-07 | 0.02424 | -1.40 | 0.43 | 1.22E-03 | 0.00279 |
| cg06913365 | -2.66 | 0.54 | 7.99E-07 | 0.02424 | -1.73 | 0.72 | 1.67E-02 | 0.02502 |
| *cg09560533* | *1.59* | 0.32 | *9.31E-07* | *0.02424* | *0.74* | 0.51 | *1.50E-01* | *0.14990* |
| cg09062708 | -1.50 | 0.31 | 9.33E-07 | 0.02424 | -1.41 | 0.44 | 1.39E-03 | 0.00286 |
| cg11932091 | -2.40 | 0.49 | 1.07E-06 | 0.02424 | -1.59 | 0.73 | 2.91E-02 | 0.03695 |
| cg05076365 | -1.76 | 0.36 | 1.14E-06 | 0.02452 | -0.99 | 0.46 | 3.04E-02 | 0.03712 |
| *cg19418235* | *-1.57* | 0.32 | *1.21E-06* | *0.02498* | *-0.81* | 0.41 | *4.91E-02* | *0.05590* |
| cg08976687 | -1.68 | 0.35 | 1.34E-06 | 0.02550 | -1.32 | 0.41 | 1.27E-03 | 0.00279 |
| cg04999580 | -2.31 | 0.48 | 1.39E-06 | 0.02550 | -2.42 | 0.62 | 8.75E-05 | 0.00048 |
| cg04468927 | -1.59 | 0.33 | 1.41E-06 | 0.02550 | -1.60 | 0.47 | 6.89E-04 | 0.00189 |
| cg03355952 | -1.81 | 0.38 | 1.51E-06 | 0.02634 | -1.87 | 0.50 | 1.68E-04 | 0.00069 |
| *cg13717333* | *-2.10* | 0.44 | *1.57E-06* | *0.02639* | *-1.17* | 0.60 | *5.11E-02* | *0.05616* |
| cg27648858 | -1.46 | 0.31 | 1.91E-06 | 0.03080 | -1.66 | 0.34 | 1.02E-06 | 0.00003 |
| *cg26385256* | *-1.89* | 0.40 | *2.12E-06* | *0.03311* | *-0.88* | 0.52 | *9.24E-02* | *0.09532* |
| cg10020385 | -3.20 | 0.68 | 2.20E-06 | 0.03311 | -2.38 | 0.87 | 6.04E-03 | 0.00996 |
| cg02843332 | 1.43 | 0.30 | 2.44E-06 | 0.03561 | 0.83 | 0.39 | 3.27E-02 | 0.03852 |
| cg02134355 | 1.62 | 0.35 | 2.65E-06 | 0.03748 | 1.33 | 0.47 | 4.91E-03 | 0.00852 |
| cg14798653 | -1.33 | 0.28 | 3.24E-06 | 0.04441 | -0.85 | 0.38 | 2.72E-02 | 0.03584 |

**Supplementary Table 1**

**Comparison of results from main versus sensitivity analysis controlling for first-degree relative history of ADHD**

**Supplementary Table 2**

**Comparison of results from main versus sensitivity analysis controlling for maternal prenatal smoking**

|  | ***Main Model*** | | | | ***Sensitivity Analysis***  ***(Maternal prenatal smoking)*** | | | |
| --- | --- | --- | --- | --- | --- | --- | --- | --- |
| **CpG** | **Coefficient** | **Std Error** | ***p* value (raw)** | ***p* value (FDR)** | **Coefficient** | **Std Error** | ***p* value (raw)** | ***p* value (FDR)** |
| cg01132150 | -1.98 | 0.34 | 4.26E-09 | 0.00193 | -2.03 | 0.34 | 1.48E-09 | 4.88E-08 |
| cg21415305 | -1.81 | 0.31 | 9.66E-09 | 0.00219 | -1.81 | 0.31 | 9.71E-09 | 1.07E-07 |
| cg09297702 | -1.83 | 0.32 | 1.85E-08 | 0.00280 | -1.89 | 0.32 | 5.06E-09 | 8.35E-08 |
| cg20139664 | -1.73 | 0.33 | 1.66E-07 | 0.01878 | -1.77 | 0.33 | 8.33E-08 | 6.87E-07 |
| cg18773807 | -2.42 | 0.48 | 3.86E-07 | 0.02003 | -2.41 | 0.48 | 5.34E-07 | 9.85E-07 |
| cg22727761 | -2.12 | 0.41 | 2.61E-07 | 0.02003 | -2.11 | 0.41 | 3.32E-07 | 9.85E-07 |
| cg08220278 | -1.55 | 0.31 | 3.98E-07 | 0.02003 | -1.58 | 0.30 | 2.10E-07 | 9.85E-07 |
| cg05182265 | -2.97 | 0.59 | 3.72E-07 | 0.02003 | -2.95 | 0.59 | 6.51E-07 | 1.03E-06 |
| cg25109393 | -2.08 | 0.40 | 2.91E-07 | 0.02003 | -2.08 | 0.41 | 3.92E-07 | 9.85E-07 |
| cg26076948 | 1.70 | 0.34 | 6.15E-07 | 0.02353 | 1.71 | 0.34 | 3.82E-07 | 9.85E-07 |
| cg01807408 | 2.18 | 0.44 | 5.69E-07 | 0.02353 | 2.15 | 0.43 | 7.84E-07 | 1.18E-06 |
| cg01277890 | -2.29 | 0.46 | 6.24E-07 | 0.02353 | -2.34 | 0.46 | 3.58E-07 | 9.85E-07 |
| cg10457436 | -1.74 | 0.36 | 9.54E-07 | 0.02424 | -1.87 | 0.36 | 1.81E-07 | 9.85E-07 |
| cg22514284 | -1.44 | 0.29 | 1.05E-06 | 0.02424 | -1.44 | 0.30 | 1.17E-06 | 1.44E-06 |
| cg12228863 | 2.44 | 0.50 | 8.30E-07 | 0.02424 | 2.51 | 0.50 | 5.37E-07 | 9.85E-07 |
| cg11237284 | -1.67 | 0.34 | 9.88E-07 | 0.02424 | -1.70 | 0.34 | 5.31E-07 | 9.85E-07 |
| cg06913365 | -2.66 | 0.54 | 7.99E-07 | 0.02424 | -2.70 | 0.54 | 6.06E-07 | 1.03E-06 |
| cg09560533 | 1.59 | 0.32 | 9.31E-07 | 0.02424 | 1.63 | 0.32 | 4.20E-07 | 9.85E-07 |
| cg09062708 | -1.50 | 0.31 | 9.33E-07 | 0.02424 | -1.54 | 0.31 | 6.58E-07 | 1.03E-06 |
| cg11932091 | -2.40 | 0.49 | 1.07E-06 | 0.02424 | -2.40 | 0.49 | 1.17E-06 | 1.44E-06 |
| cg05076365 | -1.76 | 0.36 | 1.14E-06 | 0.02452 | -1.88 | 0.36 | 1.74E-07 | 9.85E-07 |
| cg19418235 | -1.57 | 0.32 | 1.21E-06 | 0.02498 | -1.59 | 0.33 | 1.03E-06 | 1.41E-06 |
| cg08976687 | -1.68 | 0.35 | 1.34E-06 | 0.02550 | -1.75 | 0.36 | 1.20E-06 | 1.44E-06 |
| cg04999580 | -2.31 | 0.48 | 1.39E-06 | 0.02550 | -2.32 | 0.48 | 1.67E-06 | 1.83E-06 |
| cg04468927 | -1.59 | 0.33 | 1.41E-06 | 0.02550 | -1.56 | 0.33 | 2.94E-06 | 3.03E-06 |
| cg03355952 | -1.81 | 0.38 | 1.51E-06 | 0.02634 | -1.92 | 0.38 | 4.11E-07 | 9.85E-07 |
| cg13717333 | -2.10 | 0.44 | 1.57E-06 | 0.02639 | -2.13 | 0.43 | 8.45E-07 | 1.21E-06 |
| cg27648858 | -1.46 | 0.31 | 1.91E-06 | 0.03080 | -1.47 | 0.32 | 3.36E-06 | 3.36E-06 |
| cg26385256 | -1.89 | 0.40 | 2.12E-06 | 0.03311 | -1.89 | 0.40 | 1.86E-06 | 1.98E-06 |
| cg10020385 | -3.20 | 0.68 | 2.20E-06 | 0.03311 | -3.25 | 0.67 | 1.22E-06 | 1.44E-06 |
| cg02843332 | 1.43 | 0.30 | 2.44E-06 | 0.03561 | 1.54 | 0.31 | 5.18E-07 | 9.85E-07 |
| cg02134355 | 1.62 | 0.35 | 2.65E-06 | 0.03748 | 1.75 | 0.34 | 3.39E-07 | 9.85E-07 |
| cg14798653 | -1.33 | 0.28 | 3.24E-06 | 0.04441 | -1.35 | 0.28 | 1.53E-06 | 1.74E-06 |

**Supplementary Table 3**

**Comparison of results from main versus sensitivity analysis controlling for maternal low socioeconomic status (Hollingshead level 5)**

|  | ***Main Model*** | | | | ***Sensitivity Analysis***  ***(Low socioeconomic status)*** | | | |
| --- | --- | --- | --- | --- | --- | --- | --- | --- |
| **CpG** | **Coefficient** | **Std Error** | ***p* value (raw)** | ***p* value (FDR)** | **Coefficient** | **Std Error** | ***p* value (raw)** | ***p* value (FDR)** |
| cg01132150 | -1.98 | 0.34 | 4.26E-09 | 0.00193 | -1.97 | 0.33 | 4.31E-09 | 9.38E-08 |
| cg21415305 | -1.81 | 0.31 | 9.66E-09 | 0.00219 | -1.82 | 0.32 | 8.52E-09 | 9.38E-08 |
| cg09297702 | -1.83 | 0.32 | 1.85E-08 | 0.00280 | -1.88 | 0.32 | 6.81E-09 | 9.38E-08 |
| cg20139664 | -1.73 | 0.33 | 1.66E-07 | 0.01878 | -1.70 | 0.33 | 2.66E-07 | 1.43E-06 |
| cg18773807 | -2.42 | 0.48 | 3.86E-07 | 0.02003 | -2.38 | 0.48 | 7.45E-07 | 1.76E-06 |
| cg22727761 | -2.12 | 0.41 | 2.61E-07 | 0.02003 | -2.11 | 0.41 | 3.07E-07 | 1.43E-06 |
| cg08220278 | -1.55 | 0.31 | 3.98E-07 | 0.02003 | -1.56 | 0.31 | 3.48E-07 | 1.43E-06 |
| cg05182265 | -2.97 | 0.59 | 3.72E-07 | 0.02003 | -3.03 | 0.58 | 2.05E-07 | 1.35E-06 |
| cg25109393 | -2.08 | 0.40 | 2.91E-07 | 0.02003 | -2.13 | 0.40 | 1.47E-07 | 1.21E-06 |
| cg26076948 | 1.70 | 0.34 | 6.15E-07 | 0.02353 | 1.62 | 0.34 | 1.76E-06 | 2.55E-06 |
| cg01807408 | 2.18 | 0.44 | 5.69E-07 | 0.02353 | 2.15 | 0.45 | 1.78E-06 | 2.55E-06 |
| cg01277890 | -2.29 | 0.46 | 6.24E-07 | 0.02353 | -2.24 | 0.46 | 1.38E-06 | 2.38E-06 |
| cg10457436 | -1.74 | 0.36 | 9.54E-07 | 0.02424 | -1.73 | 0.36 | 1.32E-06 | 2.38E-06 |
| cg22514284 | -1.44 | 0.29 | 1.05E-06 | 0.02424 | -1.47 | 0.30 | 7.00E-07 | 1.76E-06 |
| cg12228863 | 2.44 | 0.50 | 8.30E-07 | 0.02424 | 2.39 | 0.51 | 2.64E-06 | 3.01E-06 |
| cg11237284 | -1.67 | 0.34 | 9.88E-07 | 0.02424 | -1.66 | 0.34 | 1.39E-06 | 2.38E-06 |
| cg06913365 | -2.66 | 0.54 | 7.99E-07 | 0.02424 | -2.57 | 0.55 | 2.48E-06 | 2.98E-06 |
| cg09560533 | 1.59 | 0.32 | 9.31E-07 | 0.02424 | 1.61 | 0.33 | 1.44E-06 | 2.38E-06 |
| cg09062708 | -1.50 | 0.31 | 9.33E-07 | 0.02424 | -1.53 | 0.31 | 6.72E-07 | 1.76E-06 |
| cg11932091 | -2.40 | 0.49 | 1.07E-06 | 0.02424 | -2.44 | 0.49 | 6.18E-07 | 1.76E-06 |
| cg05076365 | -1.76 | 0.36 | 1.14E-06 | 0.02452 | -1.85 | 0.37 | 4.38E-07 | 1.61E-06 |
| cg19418235 | -1.57 | 0.32 | 1.21E-06 | 0.02498 | -1.60 | 0.32 | 6.01E-07 | 1.76E-06 |
| cg08976687 | -1.68 | 0.35 | 1.34E-06 | 0.02550 | -1.67 | 0.35 | 1.55E-06 | 2.44E-06 |
| cg04999580 | -2.31 | 0.48 | 1.39E-06 | 0.02550 | -2.28 | 0.48 | 2.52E-06 | 2.98E-06 |
| cg04468927 | -1.59 | 0.33 | 1.41E-06 | 0.02550 | -1.57 | 0.33 | 2.01E-06 | 2.67E-06 |
| cg03355952 | -1.81 | 0.38 | 1.51E-06 | 0.02634 | -1.83 | 0.38 | 1.09E-06 | 2.25E-06 |
| cg13717333 | -2.10 | 0.44 | 1.57E-06 | 0.02639 | -2.07 | 0.44 | 2.02E-06 | 2.67E-06 |
| cg27648858 | -1.46 | 0.31 | 1.91E-06 | 0.03080 | -1.53 | 0.31 | 1.08E-06 | 2.25E-06 |
| cg26385256 | -1.89 | 0.40 | 2.12E-06 | 0.03311 | -1.88 | 0.40 | 2.51E-06 | 2.98E-06 |
| cg10020385 | -3.20 | 0.68 | 2.20E-06 | 0.03311 | -3.08 | 0.68 | 6.37E-06 | 6.37E-06 |
| cg02843332 | 1.43 | 0.30 | 2.44E-06 | 0.03561 | 1.42 | 0.30 | 2.75E-06 | 3.03E-06 |
| cg02134355 | 1.62 | 0.35 | 2.65E-06 | 0.03748 | 1.61 | 0.35 | 4.28E-06 | 4.56E-06 |
| cg14798653 | -1.33 | 0.28 | 3.24E-06 | 0.04441 | -1.31 | 0.29 | 4.58E-06 | 4.72E-06 |

**Supplementary Table 4**

**Comparison of results from main versus sensitivity analysis controlling for child birthweight**

|  | ***Main Model*** | | | | ***Sensitivity Analysis***  ***(Child birthweight)*** | | | |
| --- | --- | --- | --- | --- | --- | --- | --- | --- |
| **CpG** | **Coefficient** | **Std Error** | ***p* value (raw)** | ***p* value (FDR)** | **Coefficient** | **Std Error** | ***p* value (raw)** | ***p* value (FDR)** |
| cg01132150 | -1.98 | 0.34 | 4.26E-09 | 0.00193 | -1.96 | 0.33 | 3.09E-09 | 8.03E-08 |
| cg21415305 | -1.81 | 0.31 | 9.66E-09 | 0.00219 | -1.80 | 0.31 | 7.30E-09 | 8.03E-08 |
| cg09297702 | -1.83 | 0.32 | 1.85E-08 | 0.00280 | -1.86 | 0.32 | 6.07E-09 | 8.03E-08 |
| cg20139664 | -1.73 | 0.33 | 1.66E-07 | 0.01878 | -1.71 | 0.33 | 1.37E-07 | 1.13E-06 |
| cg18773807 | -2.42 | 0.48 | 3.86E-07 | 0.02003 | -2.42 | 0.48 | 3.54E-07 | 1.75E-06 |
| cg22727761 | -2.12 | 0.41 | 2.61E-07 | 0.02003 | -2.07 | 0.41 | 5.48E-07 | 1.81E-06 |
| cg08220278 | -1.55 | 0.31 | 3.98E-07 | 0.02003 | -1.52 | 0.30 | 5.34E-07 | 1.81E-06 |
| cg05182265 | -2.97 | 0.59 | 3.72E-07 | 0.02003 | -2.89 | 0.59 | 9.71E-07 | 2.00E-06 |
| cg25109393 | -2.08 | 0.40 | 2.91E-07 | 0.02003 | -2.04 | 0.40 | 3.85E-07 | 1.75E-06 |
| cg26076948 | 1.70 | 0.34 | 6.15E-07 | 0.02353 | 1.67 | 0.34 | 8.25E-07 | 1.97E-06 |
| cg01807408 | 2.18 | 0.44 | 5.69E-07 | 0.02353 | 2.18 | 0.43 | 3.77E-07 | 1.75E-06 |
| cg01277890 | -2.29 | 0.46 | 6.24E-07 | 0.02353 | -2.24 | 0.46 | 1.05E-06 | 2.04E-06 |
| cg10457436 | -1.74 | 0.36 | 9.54E-07 | 0.02424 | -1.67 | 0.35 | 1.91E-06 | 2.98E-06 |
| cg22514284 | -1.44 | 0.29 | 1.05E-06 | 0.02424 | -1.43 | 0.29 | 9.17E-07 | 2.00E-06 |
| cg12228863 | 2.44 | 0.50 | 8.30E-07 | 0.02424 | 2.50 | 0.49 | 4.24E-07 | 1.75E-06 |
| cg11237284 | -1.67 | 0.34 | 9.88E-07 | 0.02424 | -1.68 | 0.34 | 8.34E-07 | 1.97E-06 |
| cg06913365 | -2.66 | 0.54 | 7.99E-07 | 0.02424 | -2.66 | 0.54 | 6.91E-07 | 1.90E-06 |
| cg09560533 | 1.59 | 0.32 | 9.31E-07 | 0.02424 | 1.59 | 0.32 | 6.34E-07 | 1.90E-06 |
| cg09062708 | -1.50 | 0.31 | 9.33E-07 | 0.02424 | -1.45 | 0.31 | 1.99E-06 | 2.98E-06 |
| cg11932091 | -2.40 | 0.49 | 1.07E-06 | 0.02424 | -2.33 | 0.50 | 3.16E-06 | 3.66E-06 |
| cg05076365 | -1.76 | 0.36 | 1.14E-06 | 0.02452 | -1.68 | 0.36 | 3.40E-06 | 3.74E-06 |
| cg19418235 | -1.57 | 0.32 | 1.21E-06 | 0.02498 | -1.50 | 0.32 | 2.74E-06 | 3.35E-06 |
| cg08976687 | -1.68 | 0.35 | 1.34E-06 | 0.02550 | -1.63 | 0.34 | 2.17E-06 | 3.07E-06 |
| cg04999580 | -2.31 | 0.48 | 1.39E-06 | 0.02550 | -2.24 | 0.47 | 2.30E-06 | 3.07E-06 |
| cg04468927 | -1.59 | 0.33 | 1.41E-06 | 0.02550 | -1.53 | 0.33 | 3.21E-06 | 3.66E-06 |
| cg03355952 | -1.81 | 0.38 | 1.51E-06 | 0.02634 | -1.78 | 0.38 | 2.42E-06 | 3.07E-06 |
| cg13717333 | -2.10 | 0.44 | 1.57E-06 | 0.02639 | -2.07 | 0.44 | 2.38E-06 | 3.07E-06 |
| cg27648858 | -1.46 | 0.31 | 1.91E-06 | 0.03080 | -1.48 | 0.30 | 1.14E-06 | 2.10E-06 |
| cg26385256 | -1.89 | 0.40 | 2.12E-06 | 0.03311 | -1.86 | 0.40 | 3.92E-06 | 4.17E-06 |
| cg10020385 | -3.20 | 0.68 | 2.20E-06 | 0.03311 | -3.21 | 0.67 | 1.85E-06 | 2.98E-06 |
| cg02843332 | 1.43 | 0.30 | 2.44E-06 | 0.03561 | 1.46 | 0.31 | 1.68E-06 | 2.92E-06 |
| cg02134355 | 1.62 | 0.35 | 2.65E-06 | 0.03748 | 1.58 | 0.35 | 4.90E-06 | 5.05E-06 |
| cg14798653 | -1.33 | 0.28 | 3.24E-06 | 0.04441 | -1.26 | 0.28 | 8.65E-06 | 8.65E-06 |

**Supplemental Figure 1**

**QQ Plot and Inflation Statistics for Main Analysis**


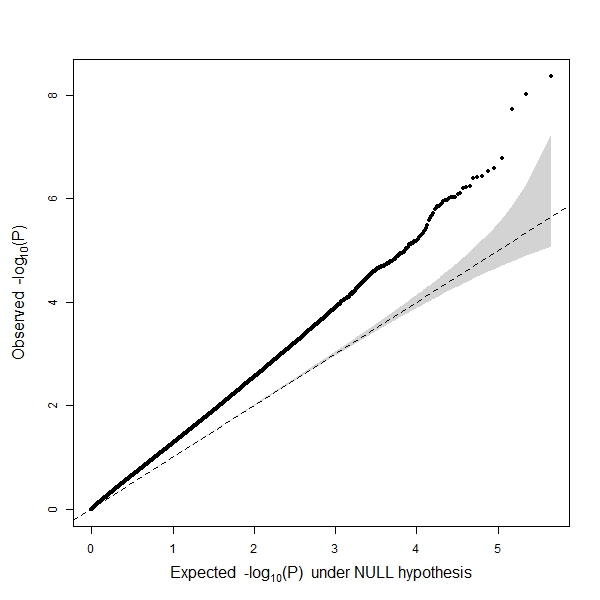


**Genomic Inflation Factor = 1.57**

**Bacon Inflation = 0.86**
